# Using qualitative mixed methods to study small health care organizations while maximising trustworthiness and authenticity

**DOI:** 10.1186/s12913-014-0559-4

**Published:** 2014-11-19

**Authors:** Christine B Phillips, Kathryn Dwan, Julie Hepworth, Christopher Pearce, Sally Hall

**Affiliations:** Medical School, Peter Baume Building 42A, Australian National University, Canberra, ACT 2601 Australia; Australian Primary Health Care Research Institute, Australian National University, Canberra, Australia; School of Public Health and Social Work, Queensland University of Technology, Brisbane, Australia; Inner East Medicare Local, Burwood, Victoria Australia; Medical School, Australian National University, Canberra, Australia

**Keywords:** Primary health care, Organizations, Qualitative research, Qualitative analysis, Mixed method research

## Abstract

**Background:**

The primary health care sector delivers the majority of health care in western countries through small, community-based organizations. However, research into these healthcare organizations is limited by the time constraints and pressure facing them, and the concern by staff that research is peripheral to their work. We developed Q-RARA—Qualitative Rapid Appraisal, Rigorous Analysis—to study small, primary health care organizations in a way that is efficient, acceptable to participants and methodologically rigorous.

**Methods:**

Q-RARA comprises a site visit, semi-structured interviews, structured and unstructured observations, photographs, floor plans, and social scanning data. Data were collected over the course of one day per site and the qualitative analysis was integrated and iterative.

**Results:**

We found Q-RARA to be acceptable to participants and effective in collecting data on organizational function in multiple sites without disrupting the practice, while maintaining a balance between speed and trustworthiness.

**Conclusions:**

The Q-RARA approach is capable of providing a richly textured, rigorous understanding of the processes of the primary care practice while also allowing researchers to develop an organizational perspective. For these reasons the approach is recommended for use in small-scale organizations both within and outside the primary health care sector.

## Background

The primary health care sector delivers the majority of health care in western countries through small community-based organizations. In Australia, the vast majority of primary care is delivered through general practice. These small, busy organizations, composed of shifting configurations of staff, vary according to their local contexts, their size, their modes of funding, and the types of services they provide. To understand the processes that underpin the delivery of quality primary care, we need to have a nuanced idea of how individuals work within these organizations. But the methodological toolkit to collect and analyse data on staff and organizational function and activities is rather limited. Much research into primary care has relied on quantitative, or single-method qualitative research. Although the importance of qualitative research methods in primary care has been emphasized since the mid-1990s, spearheaded by the *British Medical Journal* [1-3], studies typically focus on interviews or focus groups as the primary data source. Single-method studies do not capture the richness and variety of organizational functioning, the ways that staff members interact and use their time, and the impact of the spatial environment of the organization on their work.

In this paper we present an overview of a mixed-methods approach to researching small-scale primary care organizations that is rapid and rigorous (Q-RARA - Qualitative Rapid Appraisal, Rigorous Approach). We developed this method to study nurses in general practice in Australia, at a time when nurses were moving into general practice in large numbers [4]. We wished to understand the impacts of individual features of the practice, of the nurses themselves, of the other staff and of professional culture on the nurses’ roles and work. Ethnography offered one route to study emerging practices and actions within a small organizational culture, but we were concerned that the length of time to conduct organizational ethnography would mean that we would only be able to study a few sites. Q-RARA is a methodological design that is fast, flexible, and capable of producing a wide range of data from different perspectives with minimal disruption of the organization. Since conducting the Australian General Practice Nurses Study we have modified and streamlined the research design. We believe that Q-RARA is likely to be useful for research into other small scale organizations, and for bounded areas of large health organizations.

Several reasons have been put forward to explain the reliance on single-method studies, mainly interviews, focus groups and surveys in primary care research. As a rule, general practices are small businesses with few staff members all working under strict time constraints and pressure [5]. Because of the competing demands for time in general practice, staff may view research as peripheral to the purpose of the organization [6]. Under these circumstances, researchers may choose methods that can be conducted out of work hours or away from the organization.

An additional challenge for researchers is the diversity of primary care organizations in terms of structure, funding, and function within and between countries [7]. Such plurality may make inference problematic if research is based on a limited number of field-sites.

Q-RARA draws on two main influences: rapid appraisal and qualitative mixed methods research design (or QUAL-*qual* methods). The approach takes into account the need to minimize the impact of conducting research in small organizations, while maximizing the capacity to produce rich, detailed contextual findings. In this article we present: (1) an overview of the background to the approach — rapid appraisal and qualitative mixed methods design (2) the approach itself, and (3) a critical discussion of the broader literature regarding mixed methods design and issues of rigor. We identify the strengths and weaknesses of the approach, as well as its potential contribution to researching primary care organizations by extending the use of a qualitative mixed methods design.

### Antecedents of Q-RARA

#### Rapid appraisal

Rapid appraisal was pioneered in the 1960s in the field of rural studies [8], but not used systematically in health-related fields until the 1980s when rapid social science data were collected by trained [9] and, more controversially, untrained [10] researchers, on illness profiles, the understanding of disease terms, and health practices. A parallel endeavour had existed for some time in public health, where communicable disease epidemiologists used quantitative rapid appraisal methods to investigate disease outbreaks [11].

Rapid appraisal has since been used in many other settings, including humanitarian crises, and its variations are now largely referred to under the banner of Rapid Evaluation and Assessment Methods (REAM) [12]. REAM is an umbrella term that offers little detail about the processes involved in implementing a rapid appraisal approach, particularly using qualitative mixed methods. The epistemological underpinnings of REAM range from realist, objectivist epistemology (as in studies used to provide quick assessments of program performance [13]) to constructionism (in studies used to assess the impacts of policy [14] or roles [15]). The epistemological diversity of these studies reflects an under-theorization of methodology in this research area.

#### Qualitative mixed methods

The other antecedent for our method is mixed methods research. Mixed methods research is research that is informed by and situated along a spectrum of quantitative through to qualitative paradigms, and generally refers to the combination of quantitative and qualitative methods carried out “for the broad purposes of breadth and depth of understanding and corroboration” [16]. It is not new in general practice, having first been advocated over thirty years ago [17,18]. One of the caveats of this method in primary care, however, is that it can prove time-consuming and overwhelming for small institutions [19].

A particular strength of mixed methods research is the formal integration of individual methods at some point in the research process [20]. Although integration produces a more detailed, richer understanding of the phenomena of interest, in health care research integration is frequently neglected [21]. Furthermore, one of the pitfalls in mixed methods research is the conflation of integration with triangulation [22,23]. In his influential book *The Research Act*, Denzin [24] popularized the concept of triangulation as “the combination of methodologies in the study of the same phenomenon” (p. 291). The many variants, processes and critical debate surrounding triangulation [8,25] are beyond the purpose and scope of this article. We acknowledge the necessity to engage with the epistemological assumptions of the relative methods employed in any given mixed methods study so that issues of commensurability are addressed.

Morse [26] challenged qualitative researchers to consider “if, when and how” the use of two methods from the same paradigm, referred to as QUAL-*qual* methods, can be considered mixed methods. QUAL-*qual* denotes a core project and a supplementary project whereby the latter cannot be a stand-alone project. According to Morse [27] the data types, levels of analysis, or participant perspectives of the core and supplementary components need “to be handled differently and to be kept apart” (p 491).

Q-RARA attempts to marry the rapidity and limited intrusiveness of rapid appraisal methods with the rigour and integration of mixed methods research. In this paper, we subject the quality and rigor of Q-RARA to scrutiny through an assessment of its performance against Lincoln and Guba’s well-established frameworks of trustworthiness and authenticity [28].

## Methods

### The research setting

The research approach was developed as the main mechanism to conduct the Australian General Practice Nurses Study. The methods are presented in detail here for the first time. We focus in our discussion of the methods on the ability to collect multiple forms of data, and the challenges of synthesis and quality assurance for QUAL-*qual* studies.

The AGPNS was conducted from 2005 to 2008. During this period there was a nationwide, government-led promotion of practice nurses in Australia including the provision of direct incentives, newly funded Medicare items, and the nursing workforce more than doubled to nearly 9,000 [29]. This changing landscape of nursing resulted in a number of questions about the interplay of micro, meso and macro determinants on the role of nurses in primary care practice.

The AGPNS research questions were:

1. How do nurses operate within the structure of general practice?
2. What are the local, individual and structural factors that determine the role development of nurses in different general practice settings?
3. What contribution do practice nurses make to the safety and quality of general practice? and
4. How might the development of new models of practice nursing be facilitated?

These questions have been answered in a series of publications [4,30-35].

The approach was informed by a critical realist epistemology, which Harper [36] describes as a position held by researchers who “assume that our data can tell us about reality but they do not view this as a direct mirroring” (p. 88). Data are understood as being constructed through an engagement with reality and shaped by structures and practices [37]. We also operated from an interpretive theoretical perspective involving extensive analytic input from researchers by choosing to locate the study within the nurses’ work environments, and to interpret their actions, positions and the meanings they gave to their roles. The research goals, aims and questions drove the choice of mixed methods, and various types of data and analyses were conducted concurrently before being combined and subjected to further analyses [27]. Although there was a very small quantitative component in the AGPNS, overall, it was a rapid, field-based approach [12] and defined according to Morse [26] as a QUAL-*qual* method (Table 1).

**Table 1:** **Type and quantity of data collected from 25 family practices using the rapid QUAL**-***qual*** **method**

**Data collected**	**Count**	**Comments**
Qualitative		
Interviews with nurses	36 Mean, 41 minutes	Explored life history, working roles, understanding of teamwork, experiences of a GP nurse, interactions with others, notions of quality practice
Interviews with doctors	24 Mean, 27 minutes	Explored history of practice and working life within it, roles of nurses within the practice, views of potential nurse roles
Interviews with practice managers	22 Mean, 26.5 minutes	Explored history of practice and working life within it, roles of nurses within the practice, views of potential nurse roles
Observation of nurse activity	34 nurses; 51 hours of observation	2 separate hour-long structured observation of a nurse’s activities
Photographs of nurse-identified important working sites	35 nurses; 205 photographs	Photographs taken of important working sites identified by nurses within the Practice
Maps of practice layout	7 hand-drawn & 18 printed floor plans	These plans located the nurse’s station and other key sites identified by nurses or observed by researcher
Field notes	25	Field notes taken by researcher after each visit
Quantitative		
Summary of staff numbers & working hours	25	Questionnaire filled out by practice manager
Social scan	25	Details collected for each practice included: RRMA classification, distance from nearest acute hospital and community based services, number of regional general practices, allied health service availability, population data, and regional SES indicators such as unemployment rates

**Figure 1:**
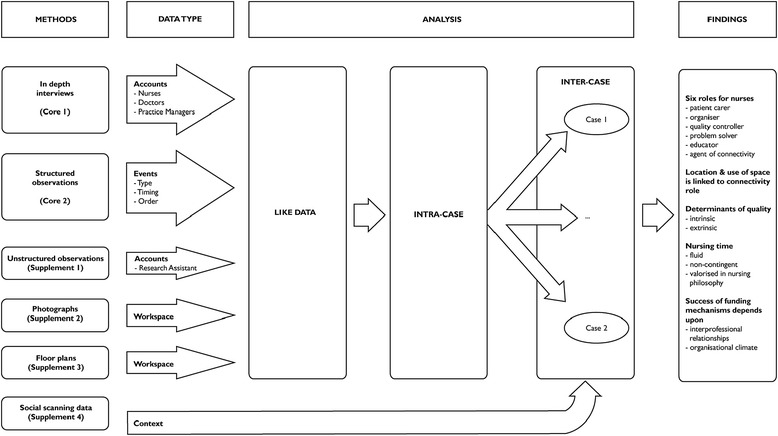
**Exploratory,** **simultaneous,** **mixed QUAL-** ***qual*** **methods.**

We collected data from 25 practices that varied in size, organizational structure and geographic location across two Australian states. Given a research setting that was undergoing rapid change and stress it was imperative that our study be undertaken with minimal disruption to health services. We modified Morse’s definition of QUAL-*qual* methods [26] by including additional methods and continued to categorize all of these as being core or supplement. These data were collected using in-depth interviews (Core 1), structured observations (Core 2), unstructured observations (Supplement 1), photographs (Supplement 2), floor plans (Supplement 3), and social scanning data (Supplement 4). The last comprised of a collation of publicly available census and health service provision data to describe the socio-geographic setting of each practice. A summary diagram of the research design is presented in Figure 1.

#### Recruitment and consent

Recruitment occurred via the then Divisions of General Practice in Victoria, NSW and the ACT, as the research team were based in Victoria and the ACT. Informed consent was obtained from both the practice as a whole for the mapping and overall observational aspects (signed by the practice principal after a briefing of the practice), and by individuals for the interviews and for the nurse observation components.

#### Ethical approval

This research was approved by the Human Research Ethics Committees of the Australian National University and the Royal Australian College of General Practitioners.

#### Data collection

The impact of the research conducted on the practices was minimized by the allocation of only one researcher to each research site and the completion of onsite data collection within one day. The two field researchers had disciplinary backgrounds in social science and community development and were not health professionals: a deliberate choice to optimize “naïve” or “fresh” observations of nurses in practices that were not obscured by prior professional experience. The field researchers scheduled the first observation early in the day because it demonstrated respect for the working hours of the practice and contributed to the development of rapport with staff. The background discussions prior to the visit and the daylong presence of a researcher meant that they were often invited to sit in on organizational activities, such as practice meetings or lunch breaks in the tearoom. The inclusion of the researchers in these ways demonstrated that the approach was acceptable to the research participants and contributed to the quality of the field notes.

#### In-depth interviews

In-depth interviews were conducted with practice staff to generate participant accounts and to assay different perspectives on the nurses’ roles. Practice nurses provided insight into their lived experience, practice managers were able to reflect on the administrative and managerial aspects of the nurses’ work, and general practitioners recounted their experiences both before and after they had decided to employ a nurse, and the impact of this decision.

#### Structured observation

The structured observation involved a small quantitative element in that observations were recorded in ten-minute lots. Some elements, such as the number of contacts with the nurse, were counted. The observations were undertaken in two one-hour periods using a paper-based system with a timer marking every ten minutes. If the practice employed more than one nurse, the field researcher attempted to observe a different nurse in the afternoon session. Preliminary trials in general practice revealed that some of the nurse contacts or activities over the period of an hour related to the same task, which were iteratively continued between other tasks (e.g. locating a missing file, providing follow-up advice for patients). The observation tool was therefore adapted to capture the cyclical nature of a nurse’s work. The trials also indicated that recording observations at intervals of less than five minutes was technically impossible for observers using a pen-and-paper observation tool unless they provided very limited details on the activities themselves. The data collection tool comprised a table in landscape format over two pages, where each row represented a 10-minute interval and each column represented a task (Table 2). The cells were completed with as much information as possible (e.g. the contents, location and participants of nurse interactions and activities). The field researchers had been briefed that the observed tasks were likely to fall into one of four categories: brief contacts, telephone calls, direct patient care, and practice administration. They were directed not to observe clinical interactions with patients, and to record the detail of them only if the nurse chose to recount to the observer what had taken place.

**Table 2:** **Example of completed structured observation**

	**TASKS**					
**TIME 9.15-10.15 am**	**Brief contacts**	**Administration**	**Supplies**	**Equipment/treatment room**	**Patient contact**	**Informal chat with research assistant**
10	Receptionist chat about problem with another staff member	Opening mail, sorting for GPs, updating records on computer	Drug rep arrives to do a free check on blood pressure monitor	Steriliser beeping to signal end of cleaning cycle. She attends to it	Checked blood pressure on elderly patient	Shows me internal mail system
20	Receptionist inquiry about arrival time of another nurse	Continues to open mail	Conversation with drug rep about next visit			Talks about other nurse’s involvement in multi-site collaborative care program
30	GP checks his pigeon hole; inquires about our project	Continues to open mail			Immunization of 8-week old baby	Chat about immunization and paperwork for recall system. Keeping track of lapsed immunizations. Her role as educator for parents about immunizations
40	Introduces GP to me	Takes patient file to GP. Enters data onto computer to be added to immunization register				
50	GP ducks in. Chat to pathology courier	Enters data on immunization onto computer			Immunization of 8-week old baby	
60				Clears plastic dishes for immunization and puts them away	Adult male of penicillin injection. 2 years old for asthma medication. Education of mother on how to use spacer device	

After a two-day training workshop, and in tandem with a chief investigator, each field researcher trialled Q-RARA to check the quality of their interview and observational skills. The concordance rate between the field researcher and the chief investigators for the time-motion studies was between 94% and 96%. As each site was visited, the data were double checked by other researchers, and the field researchers were able to debrief on the process of data collection. Many practice nurses reflected upon their activities to the researcher, resulting in a slowed-down version of the nurses’ activities over an hour. Even so, the number of activities and contacts undertaken by nurses was very high, with an average of 28 discrete activities per hour (range 6 to 60). In addition to activities, nurses sometimes moved rapidly between the consulting rooms responding to doctors, as well as communicating with patients in the waiting room. One nurse had 36 direct brief contacts with staff and patients in the hour, an average of one every 100 seconds.

#### Spatial and social contextual data

The floor plan was obtained or drawn by the observer, with patient areas and nurse workspaces marked in, and nurses were asked to identify key work areas to be photographed. The observer also collected written material from the family practice, such as practice information leaflets, and practice websites supplemented the other material. Finally, each practice was subject to social contextual mapping, which involved obtaining local demographic and contextual information about the availability of health services. Details collected for each practice included: geographical classification, distance from nearest acute hospital and community based services, number of regional family practices, allied health service availability, population data, and regional socio-economic indicators such as unemployment rates.

#### Data analysis

The researchers involved with the AGPNS had a range of disciplinary backgrounds including nursing, general practice, sociology and health policy, and they met regularly over twelve months to analyse the data and generate the findings. All data that were related to one general practice were combined to make a case. Analysis occurred in three stages: (1) All like data, such as interview data, were coded, (2) data that related to an individual general practice were examined as an *intra*-*case study*, and, (3) each practice was compared with all other practices via an *inter*-*case study*.

Interviews and field-notes were coded using NVivo 7.0 software (QSR International) by two researchers and two research assistants, employing a coding framework developed concurrently with the interview schedules. This framework was then continually revised throughout the analysis in response to research undertaken using emergent design.

Observational data were coded using a framework that distinguished tasks as administrative, clinical, servicing (e.g. stocking and sterilizing), or communicative (e.g. brief contacts with colleagues), and were then presented as pictograms. Each pictogram captured four distinct elements of the nurse’s work world: time (when), tasks (what), people (who) and locations (where), and the relationships between these elements. Producing each pictogram was an integral part of the analytic process [38]. Our pictograms functioned both as a taxonomy—an elaborated list of all the meaningful elements within the sociocultural context of a practice nurse’s work—and as a concept chart that illustrated how the key concepts of time, tasks, people and locations relate to each other temporally, processually and locationally [38]. The raw observational data were also coded into activity type and tasks funded or not funded through existing Medicare arrangements.

A researcher coded the practice floor plans and photographs using the principle that the floor plans functioned as a set for a theatrical performance, while the photographs gave insights into the way the set was used [39]. The floor plans were classified first into different zoned areas (unrestricted public areas, restricted public areas, staff-only areas, and places of clinician/patient interaction). We then catalogued the ways in which nurses moved through the practice to describe the itineraries of nurses across different spaces, comparing these with the ways other staff and patients used these spaces.

*Intra*-*case analysis*. The purpose of this level of research was synoptic, because we sought coherences across the different data types to make a finding about a particular practice, and a nurse’s role in that practice. For example, comments about space in the interviews were matched to the floor plans and photos of nurse workplaces, and were considered in the light of the time and motion studies and researcher field-notes. The intra-case analyses were returned for comment to the participant practices to establish the credibility of our generated findings [28].

*Inter-case analysis*. Two team members from different disciplinary backgrounds working independently analysed in detail the data for each emergent finding, and subsequently presented their analyses to the rest of the team for discussion. A third team member was appointed to assist with the synthesis of data and identify discrepancies in the analysis; this prevented superficial synthesis of the data, or discordant analysis. The iterative approach of the multidisciplinary team drawing on a number of data sources was able to generate a more trustworthy interpretation than would have been achieved otherwise. An example of an evolving interpretation of data from the study from a range of disciplinary perspectives is presented in Case Study 1. In Case Study 2 we set out an example of integration of like analysis, and intracase and intercase analyses.

#### Case Study 1: Evolving Insights into the Social Geography of Nurses’ Stations in General Practice

The maps of general practices showed that nurses’ stations were located either in treatment rooms or in retrofitted areas like alcoves and areas off the reception desk. There were very few nurses who had their own dedicated consultation rooms.

*Initial interpretation*. Our initial interpretation of this allocation of social space was that it reinforced the marginalization of nurses in general practice. Although it was recognized that urban practices had to retrofit their spaces to incorporate nurses, doctors on the research team felt that not having their own rooms indicated that nurses were marginalized. This view was supported on the online discussion board by some nurses.

*Second interpretation*. The sociologist wondered if the location of nurses’ rooms reflected more than professional hierarchies. She noted that nurses’ stations were usually spatially central in the practice, and co-located with the patient bed upon which the most acutely ill patients would be located. The field researchers’ notes recorded numerous instances of nurses citing the treatment room as their ideal professional locale. Review of data showed that the treatment room and the waiting rooms were the two patient-centred spaces, and that nurses visited these the most.

*Revision of second interpretation*. Field researcher reflections noted the reluctance of nurses to enter general practitioner spaces, except to find things. However, observation data and their reflections indicated that a reverse recognition was occurring with doctors reluctant to enter the treatment room without permission if it was occupied by the nurse.

*Final interpretation*. Locating the nurse’s station in the treatment room (provided the treatment room was large enough) was not seen as inappropriate or demeaning by most nurses, but rather acted as a way of reinforcing their status as parallel clinicians within the practice.

#### Case Study 2: Interpretation and Synthesis of Like, Intra-Case and Inter-Case Data

**Figure 2:**
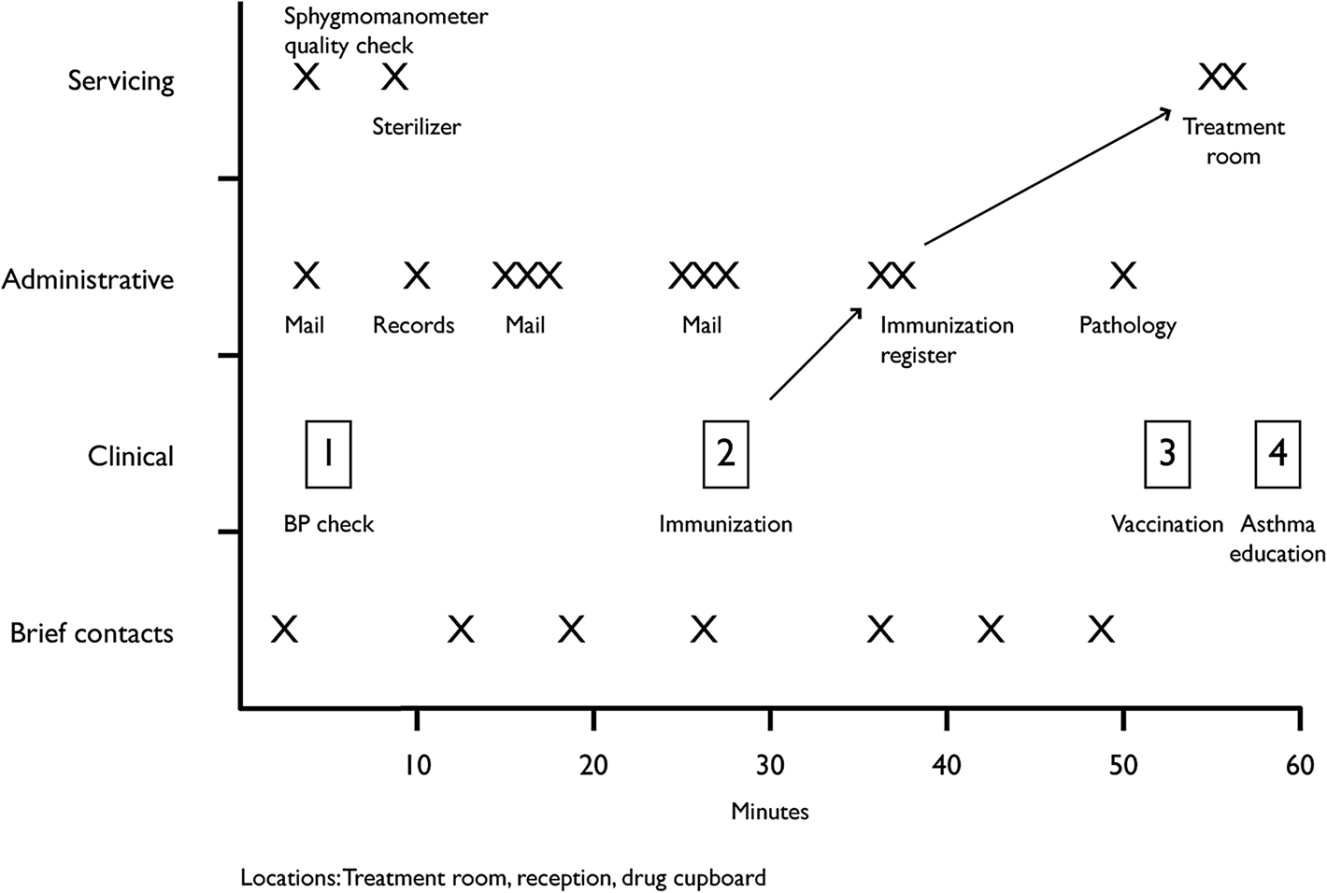
**Example of pictogram of activities undertaken by nurse in general practice.**

*Like data* (*Observation data*, *spatial mapping*, *photographs*) Table 2 presents the raw data of the *observation* of one nurses’ activities. Figure 2 is a visual synthesis of the observation data with the *spatial mapping* of the practice. During the hour she moved from the treatment room, where her desk was located, to the reception desk. Her photographs had been of staff-only places: treatment and sterilising rooms, and the drug cupboard. Her observed activities included education, clinical work and scanning and distributing of results. She had seven brief contacts (GP, receptionist, pharmaceutical representative, and courier), and solved a problem at the front desk.

*Intra*-*case synthesis* (*Synthesised from interviews with nurses*, *practice manager and doctor, social scanning and RA notes*). The nurse was new to general practice, and described herself as increasingly aware of the exigencies of small business life; hence, she had arranged for a visit from the drug rep in return for servicing the electronic blood pressure monitor. She and the GP had a collegiate relationship. He had directly recruited her from hospital to “do the things I don’t know about” (he instanced immunisations and wound dressings). The manager and the nurse had different ideas of the nursing role, with the manager wanting her to provide more services that would meet a need in the community and generate income (e. g. chronic disease clinics). The nurse described a number of educative activities - infection control, using guidelines - as key roles. The RA notes recorded that as the nurse took her photographs she commented rather ruefully on her work being in the “unseen places”.

*Inter*-*case synthesis* Comparisons across all practices were produced on lines of thought emerging from individual practices about nurses’ roles and their determinants. This case study produced as key lines of thought: “educator role”, “contribution to quality enhancement”, and “recognition”. The nurse’s workload was evolving towards education in addition to clinical work, without her employer recognising it. The drivers of connectivity identified in this study (central treatment room, permission to move across spaces, and multiple brief communications) are well-illustrated in this example. The case also illustrates the need to problematise the connectivity role. The amount of time devoted to e-connectivity may prevent the nurse from fully engaging in other work to expand her role.

## Results

We assessed the quality and rigor of the data and findings generated by Q-RARA, and interrogated its capacity to adequately reflect the stakeholders’ views, against the adequacy criteria suggested by McNall and Foster-Fishman [12] in their adaptation of the framework by Guba and Lincoln [28]. Guba and Lincoln proposed that *trustworthy* data are credible, transferable, defendable and confirmable. Data should also be assessed for *authenticity*, a domain that includes fairness, and educative, ontological, tactical and catalytic authenticity. These assessments are presented in Tables 3 and 4.

**Table 3:** **Performance of rapid QUAL**-***qual*** **Method against Guba and Lincoln**’**s trustworthiness criteria**

**Criterion**	**Definition**	**Application in research**
Credibility	Extent to which findings accurately portray respondents’ constructions. Involves the following:	
	*Prolonged engagement* in targeted site to build rapport and trust between evaluators and setting members and provide evaluators with a deeper understanding of the relevant culture.	*Prolonged engagement*: One day site visits precluded prolonged engagement.
	*Persistent observation* of site to provide sufficient understanding.	*Persistent observation*: Although it is recognized that persistent observation was not carried out for this study, the researchers attempted to respond to this criterion through repeated observation periods.
	*Peer debriefing*: Extensive discussions of data and preliminary findings with one or more peers to refine thinking.	*Peer debriefing*: RAs provided field notes and reflections for each site visit and were able to debrief with Research Manager (SH) as often as necessary. Furthermore, the questions in the qualitative interview schedule were clarified and streamlined in response to feedback from a research assistant.
	*Negative case analysis*: The constant reworking of hypotheses in light of disconfirming evidence.	*Negative case analysis*: In most practices, leadership was vested in the general practitioner, and nurses were relative newcomers to the practice. Specific analytical attention looking for difference was paid to one practice where the nurse was the senior clinician who had worked longest in the practice as a negative case.
	*Progressive subjectivity*: Researchers identify and articulate any biases they hold, examine how their understandings shift during the project, and attend to how these biases might affect interpretations.	*Progressive subjectivity*: Assumptions were regularly challenged during fortnightly analysis meetings (see Case Study 1).
	*Member checks* involve sharing and checking findings and interpretations with the people from whom the data were collected.	*Member checks*: Summaries of our research were returned to each practice for verification. We also presented our data at general practice and nursing conferences, and posted evolving data on a website developed for the project, inviting feedback from readers on the blog who were practice nurses on the summary de-identified findings and our interpretations. The Reference Group, formed at the commencement of the study, met over the course of the project and gave their feedback on topics specifically raised with them.
Transferability	Researchers describe features of targeted context in detail and suggest additional contexts to which findings might be generalized.	Extensive background and case information included in final report.
Dependability	Concerned with stability over time in researchers and methods. Assessed by means of a dependability audit, which involves reviewing project records to determine the extent to which project procedures and changes are documented.	This team included clinicians, academics and individuals engaged in organization policy and advocacy, who assisted in recruitment and in ensuring that the understanding of the project by the field sites was consistent. Regular meetings were held with all team members to monitor adherence to project procedures and to document changes in protocols.
		The three chief investigators met regularly in person and via telephone, and a summary of the decisions made were routinely produced.
Confirmability	Extent to which findings are grounded in the data. Assessed by means of confirmability audits, which involve reviewing research records to determine if findings can be traced to data and data to original sources.	Kept all case-summary, substantive theme and pattern analysis documents.
		Data and themes in all non-public documents were linked to subject IDs.
		At regular team meetings to discuss the ongoing analysis, members were encouraged to look for the “black swans”, that is, evidence that might contradict the finding under discussion.

**Table 4:** **Performance of rapid QUAL**-***qual*** **Method against Guba and Lincoln**’**s Authenticity Criteria**

**Criterion**	**Definition**	**Application in research**
Fairness	Extent to which different stakeholder perspectives are elicited and taken into account. Involves identifying all stakeholders, soliciting their perspectives, and engaging in open negotiations with them around recommendations and future actions.	We interviewed people holding a range of roles in each practice: practice nurses, a general practitioner, the practice manager and a receptionist.
		The Reference Group was particularly valuable in the final phases of the write-up, through the advice they gave on structuring the recommendations.
Ontological authenticity	Extent to which stakeholders’ perceptions of the world have been improved or expanded.	The research gave “voice” to the participants by publishing and presenting information that they knew, but was not well understood or recognized more broadly.
Educative authenticity	Extent to which individuals have developed a better understanding of other stakeholders’ experiences and perspectives.	This work was distributed in many forms (peer review journals, conference, trade press articles etc.) to both nurses and doctors, and gave support to the notion that nurses play multiple functions, some under-recognized, by general practitioners and nurses.
Catalytic authenticity	Extent to which the research elicits action and change.	There is evidence at the macro level, that previously unnoticed element of practice nursing was the extent to which nurses were “educators” and yet “doctors tended not to recognize nurses’ educator [role] … within the practice.” However, General Practice Education and Training, the national body responsible for preparing doctors for general practice, has now funded trials of practice nurses training general practice registrars.
Tactical authenticity	Extent to which stakeholders feel empowered by the evaluation and by the ability to influence the actions taken.	The findings (e.g. six roles of nurses [4]) were taken up at multiple levels by nurses from the Chief Nurse in Department of Health and Ageing to individual practice nurses. Spin-offs have included further development of some of the under-recognized roles such as the nurse as educator role through specific project funding [40].

### Trustworthiness

One of the clear challenges in assessing our approach against Guba and Lincoln’s criteria for trustworthiness was that they maintain that credible data are developed through prolonged engagement. Our method is predicated on short-term, intense engagement and cannot be compared with ethnography, which involves long-term (prolonged) engagement.

Despite their short period in the practice, research assistants were allowed access to the backstage of general practice. They were invited to observe practice meetings, accompanied nurses on their rounds when they left the practice, and followed her as she moved across all the spaces in the practice, including into doctor’s rooms. We considered that within a short-term engagement we had achieved a sense of engagement that was sufficient to meet the goals of the larger project in terms of data collection, but that this time frame may not be suitable for some other types of projects.

Against other criteria for the domain of trustworthiness the method performed well as outlined in Table 3.

#### Authenticity

The method was able to meet all the criteria contained within the domain of authenticity, as outlined in Table 4. The ability of this method to generate authentic data reflects, in part, its roots in rapid appraisal, a method that is pragmatic and purposive. On the other hand, the fact that the method also performed well against the trustworthiness criteria in general reflects the formalization of the mixed methods approach to frame rapid appraisal. An instructive comparison with our approach is research into the impact of financial incentives on clinical autonomy and internal motivation on family physicians in the UK [41]. This ethnographic study developed detailed case studies of five British family practices at a time of structural policy change, including the introduction of the National Service Frameworks and the new General Medical Services contract. The study involved in-depth, long-term contact with each of the practices. The data from this study also scored highly on both trustworthiness and authenticity criteria, but the research itself was time-consuming and the generalizability of the data in some instances was limited.

## Discussion

The approach to using Q-RARA, developed through the AGPNS, demonstrated three main features: (1) a high degree of acceptability in the field, (2) the operationalization of a modified QUAL-*qual* method, and (3) quality and rigor. First, the approach was well received by the research participants, particularly the practice nurses who believed that the interest of the researchers validated their work. In common with traditional rapid appraisal methods, Q-RARA in general practices was carried out with minimal disruption to the research site while gathering a large quantity and range of data. Feedback from the nurses collected at the time and after leaving the site, indicated that Q-RARA did not disrupt their work unduly beyond the issues described above. It also has relevance for research in other small organizations, or can be employed, as Murray [42] has demonstrated, as a mechanism for public involvement in the collection of data about social and health needs in primary care.

Second, in terms of the modification of the QUAL-*qual* method, the original definition of mixed method design proposed by Morse and Neihaus [43], p. 9 stated that it “consists of a complete method (i.e. the core component), plus one (or more) incomplete method(s) (i.e. the supplementary component[s]) that cannot be published alone, within a single study”. Morse [27] went on to specify that the data types, level of analysis or participant perspectives must be sufficiently different that they need to be handled separately prior to integration. The stipulation that one of the qualitative methods must be seen as supplemental might imply an inadequacy in the primary method, and consequently, that the need for supplemental strategies arises from “a lack of clarity on the conceptual framework of the study” [44], p. 281. In contrast, the qualitative methods used in the AGPNS were driven by the research questions and conceived prior to the site visits. Our study is not alone in this regard with many other studies in the broader health sector employing more than one complete, qualitative method [45-47].

In our approach we modified the QUAL-*qual* method by adding more than one standard qualitative method central to the study; both the in-depth interviews and the structured observation were core components. We suggest that the successful operationalization of this extended version of the QUAL-*qual* method may require alternate wording for the definition of mixed qualitative method design that addresses the concerns outlined above: QUAL-*qual* method design comprises several different qualitative methods, the choice of which is driven by the researchers’ epistemological position and theoretical perspective, and the research objectives. One or more of the methods may be able to generate sufficiently coherent and convincing findings to be published alone, but the authenticity and trustworthiness of the findings are increased by the planned use of additional research strategies that are insufficiently robust to generate defensible findings alone. The data types, level of analysis, or participant perspectives must be sufficiently different to warrant separate handling prior to integration. However, the findings generated by the different methods must be combined at some point in the research process.

The modifications to using the QUAL-*qual* method that were made in our approach are a development from existing rapid appraisal studies in primary care in that they directly engage with epistemology and integration, unlike other studies—such as Murray et al. [42,48] where the main research objective appears to be to advocate for the relevance of rapid appraisal methods in primary care, specifically interviews and focus groups or quantitative rapid appraisal methods, rather than a deeper engagement about method. Further to this, in studies where there is an exclusive qualitative mixed methods design, such as Manthorpe et al. [49] it is typical for details about individual methods to be described but a discussion of integration to be absent.

Third, the use of exploratory mixed methods provided a range of data that would have been missing had we limited ourselves to the original definition proposed by Morse [26,27]. These diverse data, in conjunction with the multidisciplinary team and an iterative approach to data analysis allowed us to engage in a dialogue with the data and produce authentic and trustworthy findings. The location of the nurse’s station as a way of reinforcing her centrality in the general practice provides an example of an evolving authentic understanding that would not have been otherwise achieved. The dialogic engagement with the data also enabled us to articulate previously unrecognized elements of the social world, that of the practice nurses’ role as agent of connectivity. The QUAL-*qual* method also revealed the nuances of social life, particularly in the way that they relate to differences in power and authority. For instance, it became apparent that the general practitioners were unaware of the range of tasks undertaken by practice nurses and their corresponding skill set [4].

The conduct of the AGPNS and development of our approach, like any field-based method, can be derailed by major events. A site visit had to be curtailed because the general practice principal collapsed and had to be taken to hospital. Nevertheless, the field researcher was able to observe and comment on the way the organization functioned in a crisis. The most difficult component of data collection was securing an interview with the general practitioner due to time pressures on their work. Consequently, some interviews with GPs were conducted by telephone after the practice visit. In small general practices, GPs have the most difficulty disposing of their time freely. Our results suggest that if this method had focused on those with least flexible time schedules in the organization, we would have had to allow more time or additional participant-responsive methods, such as video, or specific attention to informal meeting sites, such as tea-rooms.

The intensive nature of the day-long visits to practices could be draining for the field researchers, who were required to collect a great deal of data in a concentrated period of time, without disrupting the practice or losing rapport with practice staff. The two field researchers travelled very long distances to visit the sites, and were initially over-scheduled. Sufficient time between site visits is needed to collate field-notes, to recover from the trip and to prepare for the next one. Consequently, the 25 site visits were undertaken over a period of four months, equivalent to one site visit by each researcher every 10 days.

We believe that the one-day visit design is defensible even though it means that intra-clinic variation in workload across the week was not captured. All staff were asked if the observation day was typical for them, and how they structured their working week. Interviews were able to flesh out and extend the insights gained through observation; thus although there would have been differences in the observational data on different days, these did not change the broad categories of role expressions and determinants that were identified in each practice.

We noted that the structured observation tool could be improved by using more advanced technology. Nurses could be trained to use personal digital assistants or smart phone applications to record their own time use patterns. This may result in more valid data on the time use of nurses [50] but at the loss of reflections-in-action, which the field researchers then used to refine the focus of their interview schedule. Alternatively, the field researchers could continue to record the data using tablet computers.

Focus groups were not included in our version of Q-RARA because of the very small size of the health care teams, and concerns that setting up a focus group would take away too many people from the workplace. We have subsequently used them in another study using Q-RARA where we were interested in the interaction between patients and volunteer-leaders in a falls prevention program [51].

The structured observations were also limited because the researcher was not permitted to observe patient-nurse interactions. This meant that our analysis under-represented clinical care aspects of the nurse’s role. However, the fact that the nurse-patient interactions were not observed made the presence of the researcher more acceptable to the family practice itself. A method of this nature, where the researcher is only present for a short period, does not allow the researcher to develop trusting relationships with patients as, for example, an ethnographer may be able to do. To observe patient-nurse interactions would have imposed the burden of explaining the study to successive patients upon the reception and nursing staff, and slowed down the work of the practice.

## Conclusions

The dominance of interviews in qualitative general practice research typically drives researchers to explore one cadre within the practice, such as patients or healthcare providers. Consequently, there is a need for methods with proven utility that enable researchers to undertake studies of the organization itself and consider the health policy environment. We adapted and formalized REAM within a mixed-methods approach by taking the broad area of REAM and QUAL-*qual* methods, integrated these into a practical approach that is efficient, acceptable and rigorous and successfully trialled the resultant rapid QUAL-*qual* method in primary health care settings, an area that has seen little field-based research. We found our Q-RARA approach to be acceptable to participants and effective in collecting data on organizational function in multiple sites without disrupting the practice, or requiring the researcher to have a long-term presence in the practice. It was also able to strike a balance between speed and trustworthiness. Q-RARA seems capable of providing a richly textured rigorous understanding of the processes of the primary care practice while also allowing researchers to develop an organizational perspective. We have since used Q-RARA to explore community-based falls prevention activities, incorporating both movement-mapping and focus groups with patients.

For these reasons we believe the approach has merit for studying small-scale organizations both within and outside the primary health care sector, and bounded areas of larger health institutions.
